# Nanosensitizers for sonodynamic therapy for glioblastoma multiforme: current progress and future perspectives

**DOI:** 10.1186/s40779-022-00386-z

**Published:** 2022-06-09

**Authors:** Qing-Long Guo, Xing-Liang Dai, Meng-Yuan Yin, Hong-Wei Cheng, Hai-Sheng Qian, Hua Wang, Dao-Ming Zhu, Xian-Wen Wang

**Affiliations:** 1 School of Biomedical Engineering, Research and Engineering Center of Biomedical Materials, Anhui Medical University, Hefei, 230032 China; 2 Department of Neurosurgery, the First Affiliated Hospital of Anhui Medical University, Hefei, 230032 China; 3 Department of Oncology, the First Affiliated Hospital of Anhui Medical University, Hefei, 230032 China; 4 Department of General Surgery and Guangdong Provincial Key Laboratory of Precision Medicine for Gastrointestinal Tumor, Nanfang Hospital, the First School of Clinical Medicine, Southern Medical University, Guangzhou, 510515 China

**Keywords:** Glioblastoma multiforme (GBM), Blood–brain barrier (BBB), Sonodynamic therapy (SDT), Sonosensitizers, Combination therapy

## Abstract

Glioblastoma multiforme (GBM) is the most common primary malignant brain tumor, and it is associated with poor prognosis. Its characteristics of being highly invasive and undergoing heterogeneous genetic mutation, as well as the presence of the blood–brain barrier (BBB), have reduced the efficacy of GBM treatment. The emergence of a novel therapeutic method, namely, sonodynamic therapy (SDT), provides a promising strategy for eradicating tumors via activated sonosensitizers coupled with low-intensity ultrasound. SDT can provide tumor killing effects for deep-seated tumors, such as brain tumors. However, conventional sonosensitizers cannot effectively reach the tumor region and kill additional tumor cells, especially brain tumor cells. Efforts should be made to develop a method to help therapeutic agents pass through the BBB and accumulate in brain tumors. With the development of novel multifunctional nanosensitizers and newly emerging combination strategies, the killing ability and selectivity of SDT have greatly improved and are accompanied with fewer side effects. In this review, we systematically summarize the findings of previous studies on SDT for GBM, with a focus on recent developments and promising directions for future research.

## Introduction

Glioblastoma multiforme (GBM) is the most common invasive primary brain tumor, accounting for 49.1% of malignant tumors of the central nervous system (CNS). The incidence rate of GBM is also the highest among malignant CNS tumors (3.23 per 100,000 individuals) [1]. The standard treatment consists of surgery followed by adjuvant radiotherapy (RT) and/or chemotherapy [2], which results in a median overall survival (OS) of 14.6 months for patients with GBM. Despite aggressive treatment, almost all patients with GBM experienced recurrence [3]. Fewer than 6.8% of the patients can live longer than 5 years [1]. To improve the efficacy of GBM treatment, immunotherapy and physical therapy methods have been introduced in recent years. However, phase II and III clinical trials using monoclonal antibodies targeting programmed death—1 for the treatment of recurrent GBM revealed no improvement in OS [4, 5]. Tumor treating fields (TTFields) have attracted the attention of clinicians and researchers owing to their success in prolonging the OS of patients with GBM to 20.6 months [6]. Since then, the application of physical therapy for GBM has shown promise.

Ultrasound (US), which involves mechanical vibration wave of objects with strong tissue penetration ability, has been widely used in clinical applications, such as imaging and high-intensity focused US (HIFU). Among these techniques, sonodynamic therapy (SDT) is a newly emerging therapy that activates agents that have become cytotoxic upon US irradiation. Low-intensity US can activate sonosensitizers that accumulate in tumor tissues and generate reactive oxygen species (ROS) and cavitation bubbles to eradicate malignant tumor cells [7]. SDT has unique advantages, which have allowed it to achieve good results in GBM treatment. In addition, because of the poor prognosis of GBM, SDT may improve the treatment effects. Conventional sonosensitizers, such as porphyrin and its derivatives, have been used to treat GBM cells. But because of the blood–brain barrier (BBB) and the poor accumulation efficacy of sonosensitizers, ideal elimination of tumor cells cannot be achieved with SDT. Therefore, several novel strategies, including the development of multifunctional nanosonosensitizers, comprehensive nanoplatforms, and combination therapies have been developed to improve the capability of SDT. Multifunctional nanosonosensitizers generally comprise organic/inorganic sonosensitizers for ROS generation, reformative molecules for improved BBB permeability, tumor-specific agents for identification of precise tumor location, and metal ions for enhanced magnetic resonance imaging (MRI). Physical methods, such as focused US (FUS) in combination with microbubbles (MBs), can achieve temporary opening of the BBB, which aids in passing more sonosensitizers through the BBB for assembly in the intracranial tumor region. MRI imaging and thermal monitoring devices have been incorporated into the nanoplatforms for timely and precise tumor treatment. Additionally, SDT-based combination therapy can yield significant synergistic effects, which can compensate for the disadvantages of SDT and markedly enhance the efficacy of GBM treatment.

**Fig. 1:**
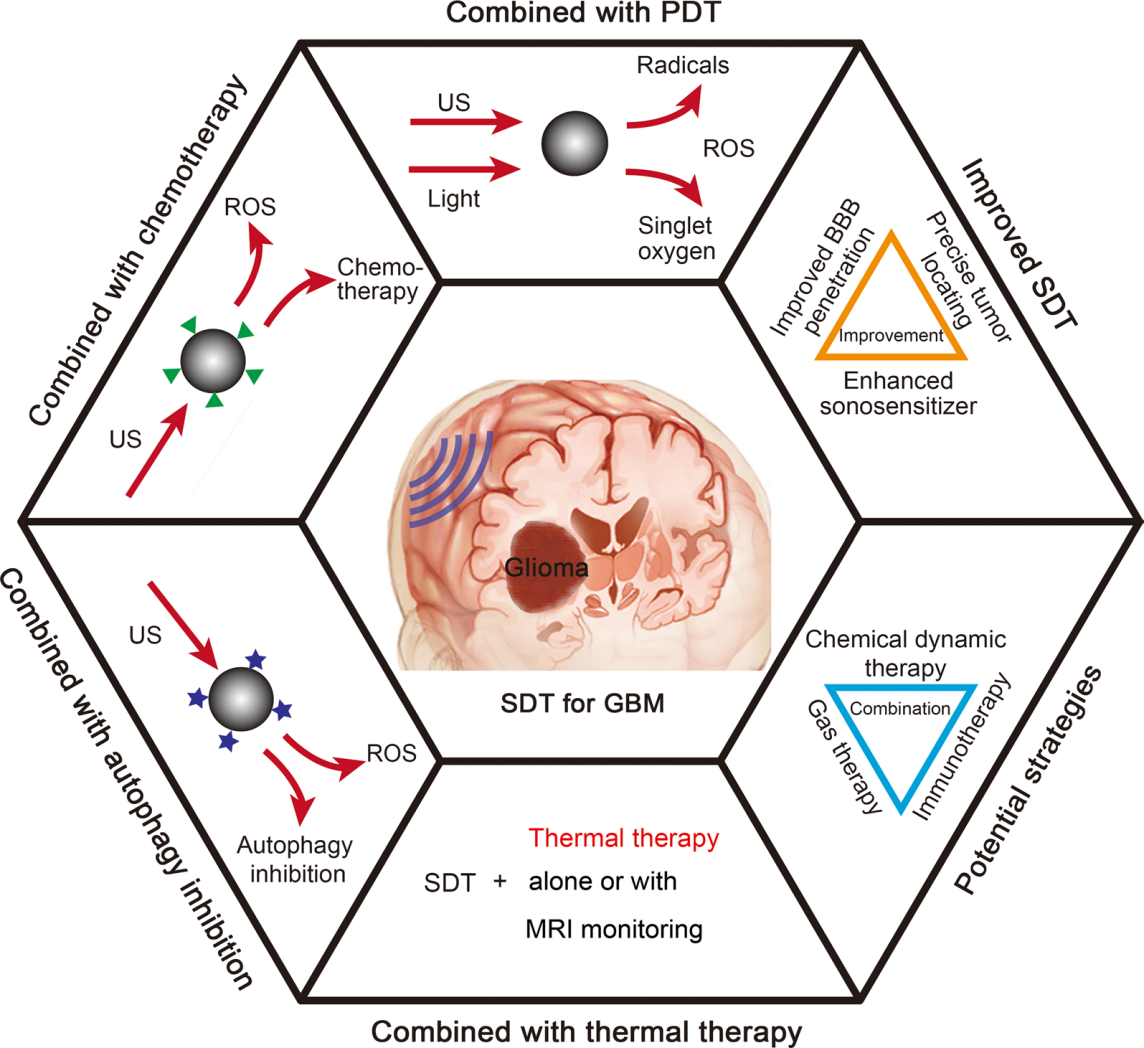
Schematic of SDT strategies for the treatment of GBM. GBM glioblastoma multiforme, PDT photodynamic therapy, ROS reactive oxygen species, SDT sonodynamic therapy, US ultrasound, MRI magnetic resonance imaging

Although many recent reviews have summarized the classification, preparation, and therapeutic application of sonosensitizers, none have provided a systematic summary of SDT for GBM [8–12]. The present review systematically summarizes the current treatment status of GBM, the mechanisms of conventional SDT, improved SDT strategies, and combination strategies (Fig. 1). The characteristics of GBM are described, followed by the advantages and disadvantages of conventional treatment strategies for GBM. Next, the application of SDT for GBM based on sonosensitizers is described in detail. In addition to these developments, combination therapies have been proposed by many researchers. Currently, several SDT-based combination treatments in GBM have been investigated, such as SDT-photodynamic therapy (PDT), SDT-chemotherapy, SDT-autophagy inhibition, and SDT-thermal therapy. These combination strategies can play a synergistic role in tumor ablation, thereby markedly enhancing the efficacy of GBM treatment. As more combination strategies have been explored in other tumor models, some of these strategies can be introduced to cure GBM, which has similar growth characteristics and tumor microenvironment (TME) alterations. For this reason, we have proposed potential combination strategies to improve the efficacy of SDT, which has broad application potential in GBM treatment. Finally, this review provides a high-level overview of the challenges and prospects of SDT. We believe that this review will indicate the direction for the future development of SDT for GBM treatment.

## Current treatment status of GBM

**Fig. 2:**
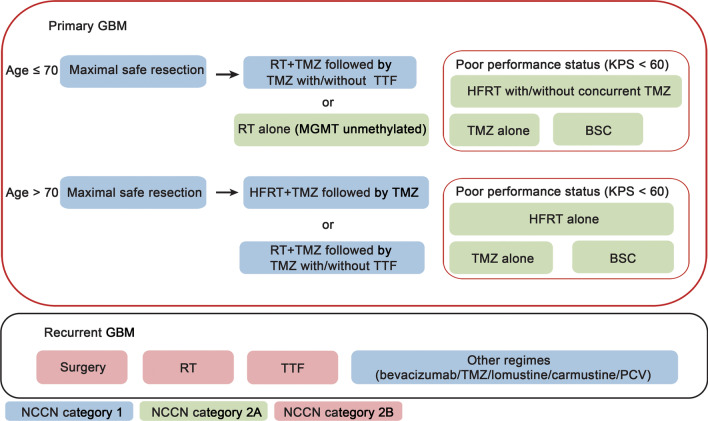
Standard treatment strategy for GBM. GBM glioblastoma multiforme, RT radiotherapy, TMZ temozolomide, TTF tumor treating fields, MGMT O^6^-methylguanine-DNA methyltransferase, HFRT hyperfractionated radiotherapy, KPS Karnofsky performance score, BSC best supportive care, PCV procarbazine, lomustine, and vincristine regimen, NCCN National Comprehensive Cancer Network

Currently, the standard approach for primary GBM is maximal safe surgical resection followed by RT (2 Gy/d, 5 d/week for 6 weeks) plus concurrent daily temozolomide (TMZ, 75 mg/m^2^), followed by 6 cycles of adjuvant TMZ (150–200 mg/m^2^, for the first 5 d/28 d per cycle) [2]. Randomized clinical trials utilized TTFields as additional effective methods in combination with TMZ to treat patients with GBM who had received standard chemoradiotherapy postoperatively [6]. Thus far, the treatment for recurrent or relapsed GBM has not been well established. Further surgical resection, re-irradiation, bevacizumab or lomustine administration, and combined approaches may be good choices. The current treatment strategy is summarized in Fig. 2 [13].

### Surgery

Surgery is the primary treatment for GBM. Maximal safe resection has been recommended by accumulating clinical evidence to achieve long-term disease control [14–16]. Currently, patients can benefit from technological advances and refinement of surgical tools, which help maximize the extent of tumor resection while minimizing morbidities. For instance, multimodal imaging integrated with preoperative MRI, diffusion tractography imaging [17], functional MRI, intraoperative MRI [18], neurophysiological monitoring [19], intraoperative US [20], and 5-aminolevulinic acid (5-ALA)-based fluorescence imaging can help neurosurgeons identify precise tumor margins and avoid damage to eloquent areas and neural fibers [21].

### RT

Since the 1960s, RT has been used as an adjuvant treatment for patients with GBM after surgery [22]. Ionizing radiation damaged the DNA of the remaining GBM tissue, which causes tumor cell death. Currently, a median OS of nearly 1 year can be achieved with RT alone [23], whereas the addition of the oral alkylating agent TMZ increases the OS to 14–16 months. The detailed chemotherapy paradigm will be discussed in “Chemotherapy” section. For patients aged < 70 years with a Karnofsky performance score (KPS) of ≥ 60, the general dose radiation scheme is 60 Gy with 2 Gy fractions over a period of 6 weeks. For elderly patients with a KPS of ≥ 50, hypofractionated RT can achieve similar effects but with increased survival period and less corticosteroid requirement than conventionally fractionated RT [22]. However, older patients with poor performance status usually have shorter OS. The prognostic information should be taken into consideration according to each individual when selecting the RT regimen.

### Chemotherapy

Chemotherapy is generally used as an adjuvant treatment for GBM. TMZ has been recommended as the first-line treatment for GBM [24]. It can induce alkylation of genomic DNA at the N^7^ and O^6^ positions of guanine and N^3^ position of adenine, resulting in a mismatched nucleotide, which can cause tumor cell death [25]. Unfortunately, the sensitivity of TMZ is largely affected by the expression of O^6^-methylguanine-DNA methyltransferase (MGMT), which can remove TMZ-induced alkylation from the nucleotides [26]. Patients without MGMT-methylated mutations tend to be less responsive to TMZ and cannot benefit from this type of treatment.

Bevacizumab is a vascular endothelial growth factor A (VEGF-A) inhibitor that suppresses angiogenesis by antagonizing VEGF-A, which can stimulate the generation of new blood vessels in tumor tissues. Considering the high expression levels of VEGF-A in GBM, bevacizumab was introduced to inhibit GBM progression. Unfortunately, it did not significantly improve the OS of patients with primary GBM [27]. Currently, it is primarily recommended for recurrent GBM, especially when it is difficult to ascertain whether postoperative neuroimaging changes are due to a radiation response or tumor recurrence.

### Immunotherapy

Immunotherapy is a type of treatment in which the suppressed immune system induced by the tumor is reactivated. Immunotherapy for the treatment of various solid cancers has resulted in great survival benefits. Consequently, many immune-based therapeutics for GBM treatment have been explored. Preclinical studies using immune checkpoint inhibitors [28], dendritic cell (DC) vaccines [29], and chimeric antigen receptor T cells have reported robust immune responses in animal models [30]. Several phase III clinical trials failed to demonstrate the corresponding OS benefits [4, 31]. These may be related to the intrinsic characteristics of GBM cells (loss of neoantigens targeting therapeutic T cells) [32] and extrinsic mechanisms (systemic immunological suppression), thereby compromising the effects of immunotherapy [31].

### Physical therapy

Physical therapy refers to the treatment of tumors by physical methods, such as sound, light, electricity, and magnetism. TTFields are electromagnetic fields in which electrical fields with low-intensity and intermediate frequency are applied to eradicate tumor cells. The application of TTFields for recurrent GBM and newly diagnosed GBM was approved by the Food and Drug Administration (FDA) in 2011 and 2015, respectively. The antimitotic effects produced by alternating electric fields can damage rapidly dividing tumor cells, leading to mitotic arrest and apoptosis [33]. A randomized clinical trial demonstrated that TTFields as an adjuvant treatment could significantly prolong the median progression-free survival from 4 months to 6.7 months and the median OS from 16 to 20.9 months [6].

Laser interstitial thermal therapy (LITT) is a technique in which thermal energy is used to treat GBM through stereotaxic guidance. An optical fiber is guided through a hole drilled with a burr in the skull to the tumor center and burns the tumor tissues using heat under MRI observation. LITT can be used to treat inoperable tumors using a minimally invasive approach. Patients with a tumor volume of < 4 cm^3^ tend to experience an OS benefit with LITT [34]. However, the swelling and inflammation effects may lead to neurological dysfunction due to the limited space of the skull. In light of improved biophysical methods, increasingly more physical methods to enhance BBB penetration are being developed, including SDT, PDT, and FUS. These approaches usher new hopes for improving the prognostic outcome of patients with GBM.

In summary, the treatment of GBM has been confronted with many obstacles and has reached the bottleneck period. Most of the effortless endeavors are owing to the existence of the BBB which can compromise the efficient delivery of drugs into the brain parenchyma. Therefore, it is necessary to review the BBB thoroughly both in physiological and pathophysiological conditions.

## Alterations in the BBB structure in brain tumors

The BBB is a highly selective semipermeable structure that prevents solutes in blood circulation from crossing into the CNS non-selectively. It is crucial for the homeostasis of the CNS and drug delivery for brain diseases. The BBB is disrupted by infiltrating tumor cells in patients with GBM, which can lead to increased penetration of the BBB and accumulation of tumor-associated immune cells, thereby altering the homeostasis of the CNS.

### Physiological and pathophysiological structure of the BBB

**Fig. 3:**
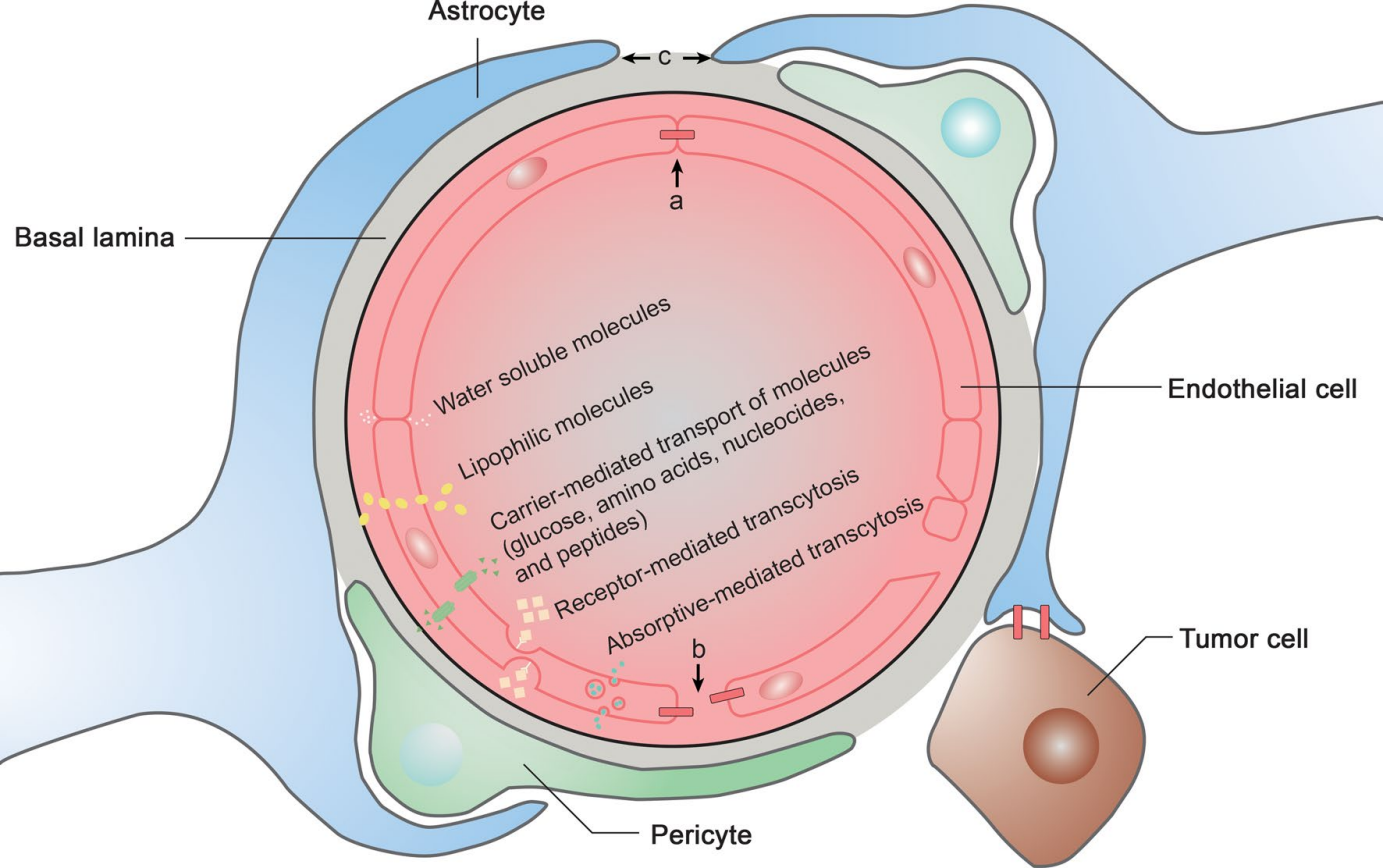
Physiological and pathophysiological structures of the blood–brain barrier (BBB). BBB structure alterations in brain tumors and types of small molecules that cross the BBB. **a** Tight junction in physiological status, < 1 nm. **b** Tight conjunction in tumor bearing status, > 7 nm. **c** Approximately 20 nm

The healthy BBB is comprised of capillary endothelial cells, the end-feet of astrocytes, and pericytes. The healthy BBB can prevent drug penetration due to the limited distance and biological characteristics of each component [35]. In patients with GBM, the heterogeneous permeability of the BBB is altered as a result of interactions of stroma-cancer cells to facilitate tumor-related immune cell infiltration and cancer cell proliferation (Fig. 3).

### Types of small molecules that cross the BBB

There are five types of molecules that cross the BBB. Water-soluble molecules can pass through the BBB via tight junctions. Lipid-soluble molecules can pass through endothelial cells via passive diffusion. Peptides are transported via carrier-mediated mechanisms. Absorptive-mediated transcytosis or endocytosis is responsible for cationic drug transportation. Last, receptor-mediated transcytosis can transport large molecules across the BBB (Fig. 3) [36].

In summary, the presence of the BBB has considerably hindered drug molecules from entering the CNS efficiently, as such, traditional agents cannot play an effective role in GBM treatment. Therefore, other strategies aimed at improving BBB permeability in GBM could improve the efficiency of drug delivery.

## Killing mechanisms of SDT

**Fig. 4:**
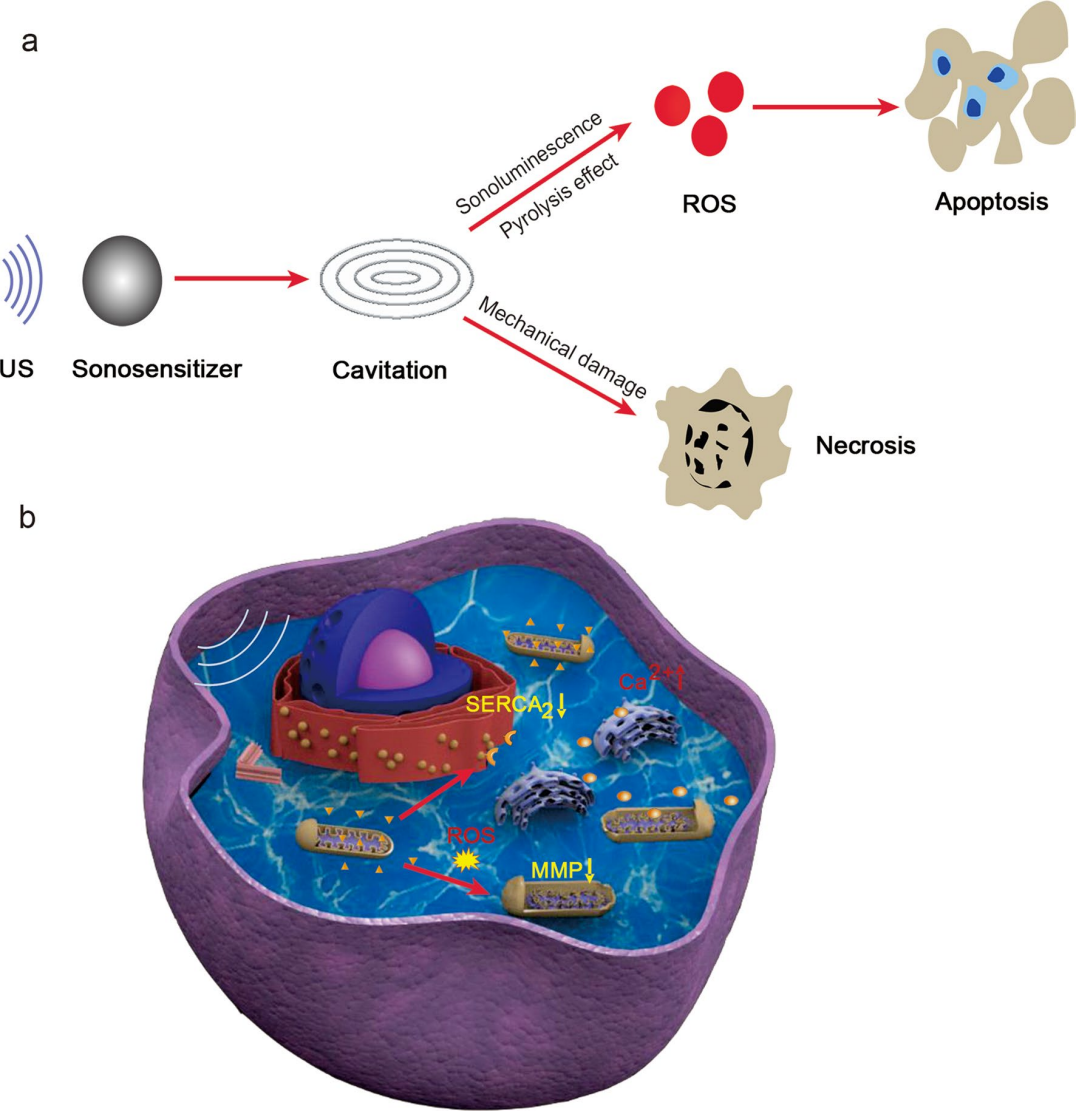
Schematic overview of the SDT mechanisms in gliomas. **a** General SDT mechanism. **b** US activation of the sonosensitizers accumulate in the mitochondria could induce the generation of ROS, which would result in the mitochondria swelling and mitochondria membrane potential (MMP) decreasing. Meanwhile, the degradation of the sarcoplasmic/endoplasmic reticulum Ca^2+^ ATPase (SERCA_2_) can lead to an abnormal increase in calcium. Both mechanisms can eventually promote apoptosis of glioma cells. SDT sonodynamic therapy, US ultrasound, ROS reactive oxygen species

US is a type of mechanical sound wave with frequencies of > 20 kHz, which is usually regarded as the upper audible limit of human hearing. With a high penetration depth of > 10 cm in soft tissues, US has been widely used as a diagnostic imaging modality for nearly 50 years to determine the size, structure, and pathological lesions of organs and tissues. Utilizing the cytotoxic effect of activated sonosensitizers by low-intensity US, tumor ablation can be achieved with SDT even if the tumor is located deeply within the body. Typically, sonosensitizers can selectively accumulate within the target tumor. Upon activation, tumor cells can be killed selectively without damage to adjacent healthy tissues. The use of SDT for the treatment of tumors was first introduced in 1990 [37]. The use of SDT for the treatment of GBM was first reported in 2008 [38]. The potential mechanism of SDT for tumor treatment has not been fully explored thus far. Traditional mechanisms have been accredited to the generation of ROS [39] and ultrasonic cavitation (Fig. 4a) [40].

### Cavitation effects

The generation of cavitation is usually promoted by sonosensitizers under US [41]. Cavitation is a complex process that is associated with gas oscillation. Gas in the aqueous solution oscillates via compression and decompression cycles under US, which can help generate microbeams and radiation forces. Cavitation can be divided into two types: inertial and non-inertial cavitation. Non-inertial cavitation causes fluid movement and promotes the mixture of surrounding media. On the other hand, inertial cavitation can result in high temperature and shock waves that can lead to mechanical damage of tumor cells [42]. Cavitation generally results in the death of a small number of cancer cells, but it is not the dominant killing mechanism.

### Generation of ROS

ROS are highly reactive chemical substances formed from oxygen (O_2_) that consist of peroxide, superoxide, hydroxyl radical, singlet oxygen, and alpha-oxygen [43]. US can activate sonosensitizers from the ground state to the excited state and release energy to subsequently generate ROS, which is generally attributed to cavitation effects. Additionally, pyrolysis and sonoluminescence can aid ROS generation, which can destroy proteins, damage DNA, and promote intracellular lipid peroxidation, resulting in cell apoptosis [10]. Generally speaking, the main killing mechanism of SDT occurs when a large amount of ROS generated by SDT causes oxidative stress to cancer cells, which induces the death of cancer cells.

### Molecular mechanisms of SDT for GBM treatment

Several studies have explored the molecular mechanisms of SDT for GBM treatment through in vitro and in vivo experiments. Many drugs, including 5-ALA [44–52], fluorescein [53], rose Bengal [54], hematoporphyrin mono-methyl ether (HMME) [38, 55–60], sino-porphyrin [61–63], photofrin [64, 65], photolon [66, 67], protoporphyrin IX (ppIX) [52], talaporfin sodium [52], aluminum phthalocyanine disulfonate (AlPcS_2a_) [68, 69], and titanium dioxide (TiO_2_), have been used as sonosensitizers [70, 71], which mainly accumulate in the mitochondria (Table 1).

**Table 1:** Summary of modality and sonosensitizers used for SDT therapy in glioblastoma

Modality	Sonosensitizers	Cell line	US power	References
Traditional SDT	5-ALA	RG2	1 MHz, 0.5 W/cm^2^, 3 min	[44]
	5-ALA	U87/U251	3 MHz, 2 W/cm^2^, 3 min	[45]
	5-ALA	C6	1.06 MHz, 0.33–8 W/cm^2^	[47]
	5-ALA	C6/U87	1.1 MHz, 10 W/cm^2^, 3 min	[48]
	5-ALA	C6	1.04 MHz, 10 W/cm^2^, 5 min	[50]
	5-ALA	C6	1 MHz, 2.65 W/cm^2^, 20/40/60 min	[51]
	Fluorescein	C6	2–6 W/cm^2^, 20 min	[53]
	Rose Bengal	C6	1 MHz, 25 W/cm^2^, 5 min	[54]
	HMME	C6	1 MHz, 0.5 W/cm^2^, 2 min	[56]
	HMME	C6	1 MHz, 1 W/cm^2^, 1 min	[55]
	HMME	C6	0.5 MHz, 1 W/cm^2^, 1 min	[58]
	HMME	C6	1 MHz, 0.5 W/cm^2^, 2 min	[38]
	HMME	C6	1 MHz, 0.5 W/cm^2^, 2 min	[59]
	HMME	C6	1 MHz, 0.5 W/cm^2^, 90 s	[60]
	DVDMS	U87	0.97 MHz, 3 min	[61]
	DVDMS	U118/U87	1 MHz, 0.5 W/cm^2^, 1 min/3 min	[62]
	DVDMS	U373	1 MHz, 0.45 W/cm^2^, 1 min	[63]
	Photofrin	GSC/U251	1 MHz, 0.5 W/cm^2^, 1 min	[64]
	Photofrin	GSC	1 MHz, 0.5 W/cm^2^, 2 min	[65]
	Photolon	C6	0.88 MHz, 0.2/0.4/0.7 W/cm^2^, 1 min	[67]
	5-ALA/PPIX/talaporfin	C6/U87	1 MHz, 0.16 W/cm^2^, 1 min	[52]
	TiO_2_	U251	1 MHz, 1 W/cm^2^, 30 s	[70]
Improved SDT	iRGD-Lipo-DVDMS	C6	1 MHz, 1 W/cm^2^, 1 min	[72]
	DVDMS-Mn-LPs	U87	0.5 MHz, 0.5 W/cm^2^, 5 min	[73]
	MnO_2_@Tf-ppIX	C6	1 MHz, 1.5 W/cm^2^, 3 min	[74]
	PpIX@HMONs-MnOxRGD	U87	1 MHz, 1.5 W/cm^2^, 1 min	[75]
Combination therapy	HPPH@PAA-NMe^3+^	U87	3.3 MHz, 0.5 W/cm^2^, 30 min	[76]
	Photolon	C6	1 MHz, 0.4/0.7/1.0 W/cm^2^, 10 min	[66]
	HMME + TMZ	C6	1 MHz, 1 W/cm^2^, 0–24 h	[57]
	AlPcS_2a_ + BLM	F98	1 MHz, 3 min	[68]
	AlPcS_2a_ + BLM	F98	1 MHz, 0–0.6 W/cm^2^, 3 min	[69]
	TiO_2_ + anti-EGFR antibody	U87MG/U87MGde2–7	1 MHz, 1.8 W/cm^2^, 1 min	[71]
	IR780/PTX	U87	1 MHz, 0.2–0.4 W/cm^2^, 3 min	[77]
	Dox-pp-lipo	U87	1 MHz, 0.3 W/cm^2^, 3 min	[78]
	ACHL	GL261	1 MHz, 1 W/cm^2^, 1 min	[79]
	5-ALA	SNB19/U87	1 MHz, 1 W/cm^2^, 2 W/cm^2^, 2 min	[46]
	5-ALA	F98	4000/500 J, 20/18 W, 240/30 s	[49]
	HCM NAs	U87	1 W/cm^2^	[80]
	5-ALA	F98	500 J, 18 W, 30 s	[81]

*5-ALA* 5-aminolevulinic acid, *HMEE* hematoporphyrin mono-methyl ether, *DVDMS* sino-porphyrin sodium, *ppIX* protoporphyrin IX, *iRGD* cyclic arginine-glycine-aspartic pentapeptide, *Tf* transferrin, *HPPH* 3-(1′-Hexyloxy) ethyl-3-devinylpyropheophorbide, *PAA-NMe*_*3*_^+^ cationic polyacrylamide nanoparticles, *TMZ* temozolomide, *AlPcS*_*2a*_ aluminum phthalocycanine disulfonate, *BLM* bleomycin, *EGFR* epithelial growth factor receptor, *PTX* paclitaxel, *DOX* doxorubicin, *Lipo* liposome, *ACHL* angiopep-2-modified liposomes, *HCM NAs* manganese ion (Mn^2+^)-chelated human serum albumin (HSA)-chlorin e6 (Ce6) nanoassemblies

Upon activation by US, ROS generation can result in a decrease in mitochondrial membrane potential (MMP) as well as mitochondrial swelling [46, 48, 54, 56–58, 61, 63]. Meanwhile, the degradation of the sarcoplasmic/endoplasmic reticulum Ca^2+^ ATPase (SERCA_2_) in the endoplasmic reticular can lead to elevation of cytoplasmic Ca^2+^ levels [56, 58]. The synergistic function causes tumor cell apoptosis (Fig. 4b).

To sum up, SDT can eradicate GBM cells mainly through ROS-induced apoptosis mediated by a decrease in MMP and elevation of intracellular Ca^2+^ levels.

## Improved SDT with multifunctional nanosonosensitizers in GBM treatment

Considering the limited permeability of the BBB and the low efficacy and accuracy of sonosensitizer accumulation, many strategies have been developed to improve the efficacy of SDT for GBM treatment, including improved BBB penetration for sonosensitizers, enhanced tumor-targeted sonosensitizer accumulation, and identification of precise tumor location, which is generally referred to as imaging—guided treatment. Many researchers have improved the efficacy of SDT for GBM treatment by manufacturing multifunctional sonosensitizers that incorporate enhanced BBB penetration, tumor-targeted accumulation, and MRI abilities.

### Enhanced BBB penetration for sonosensitizers

The limited permeability of the BBB presents a challenge for sonosensitizers to enter the CNS. The BBB allows only specific small molecules to pass through it. Although tumors can lead to increased BBB penetration, it is insufficient to meet the requirements for efficient delivery of therapeutic agents. Hence, new strategies have been introduced to overcome these disadvantages.

#### Reversible opening of the BBB using ultrasound-targeted microbubble destruction (UTMD)

First introduced in 2001, UTMD involves the combination of FUS with MBs, which can result in the noninvasive and reversible opening of the BBB [82]. MBs begin to oscillate at the frequency of US upon exposure to sonication. The stable cavitation can generate mechanical stress, which further disrupts tight junctions and increases the permeability of the BBB [83]. Nevertheless, inertial cavitation can induce MB collapse with micro-jetting, fragmentation, and shock-wave formation, which might cause damage to the vascular endothelial cells [84]. Therefore, stable cavitation (application of a few hundred kPa) is generally considered a safe approach for BBB opening [85]. Studies investigating FUS-induced drug delivery for the treatment of GBM [86], Alzheimer’s disease [87], and Parkinson’s diseases are underway [88].

#### Designation of drug delivery systems (DDSs)

More than 98% of small-molecule agents, such as peptides, recombinant proteins, monoclonal antibodies, and nucleic acids, cannot pass through the BBB. To overcome this obstacle, biomimetic DDSs featuring the mechanism of a natural nutrient supply were developed to improve the permeability of the BBB [89]. According to the modified ligands, DDSs can be generally divided into cell membrane-based DDSs, lipoprotein-based DDSs, exosome-based DDSs, virus-based DDSs, protein template-based DDSs, and peptide template-based DDSs, among which liposomes and transferrin are most widely used in SDT for GBM. The liposomes usually pass through the BBB via passive diffusion, and transferrin can improve BBB permeability through receptor-mediated endocytosis. Furthermore, DDSs can not only penetrate the BBB easily but also have good biocompatibility. In this way, the employment of agents against GBM can be maximized.

### Enhanced sonosensitizer accumulation (targeted tumor therapy)

Traditional sonosensitizers such as sinoporphyrin sodium (DVDMS) have demonstrated excellent effects of SDT on eliminating glioma cells and have been regarded as effective sonosensitizers and photosensitizers [90, 91]. However, DVDMS is a hydrophilic macromolecule that cannot pass through the BBB, which leads to its low bioavailability and tumor selectivity. Consequently, the internalizing iRGD-modified DVDMS liposome (iRGD-Lipo-DVDMS) was developed to improve BBB permeability and tumor selectivity [92]. iRGD is a tumor-homing peptide with the sequence CRGDKGPDC that has excellent tumor identification and tumor penetration ability [93]. iRGD functions through specific binding of the RGD sequence to the αvβ3 and/or αvβ5 integrins, which are typically overexpressed in tumor vessels and cells. iRGD is then hydrolyzed by host proteases, exposing the GendR motif 79, which can interact with neuropilin to promote internalization of tumor cells and tissues [72]. In the study, the median OS of orthotopically implanted C6 glioma mice treated with iRGD-Lipo-DVDMS-SDT (40 d) was significantly longer than that of those treated with only saline (15 d), free DVDMS (19 d), or Lipo-DVDMS-SDT (24 d). The body weight of the glioma-bearing mice was also highest in the iRGD-Lipo-DVDMS-SDT group, thereby showing excellent anti-glioma efficacy. Targeted tumor therapy can improve the eradication rate of GBM cells and simultaneously reduce the side effects caused by the sonosensitizers.

### Precise determination of glioblastoma location (MRI-guided cancer therapy)

Some studies reported MRI-guided SDT using the imaging function of metal ions, which can help determine the peak accumulation time and location of sonosensitizers in tumors. This can provide precise guidance on when and where to apply SDT. Liu et al. [73] encapsulated DVDMS-Mn-LPs (DVDMS chelated with manganese ions) into nanoliposomes. This type of nanosensitizer utilized chelated Mn^2+^ for contrast‐enhanced T_1_‐weighted MRI, DVDMS for efficient SDT, and liposomes for drug delivery. The T_1_ longitudinal relaxation rate (r_1_) of the DVDMS‐Mn‐LPs was four times higher than that of Gd‐based Magnevist, which was approved for clinical application. Three hours after the injection of DVDMS-Mn-LPs, the T_1_ signals of tumors in mice peaked. Thereafter, SDT (1.5 W/cm^2^, 10 min) was applied for 6 h on the orthotopic glioma mouse model after intravenous administration of DVDMS‐Mn‐LPs. Consequently, DVDMS‐Mn‐LPs-assisted SDT showed the best prognostic outcome compared with that in the PDT and control groups.

To explore the more extensive MRI function of Mn^2+^, Liang et al. [74] constructed a smart nanoplatform using holo-transferrin (holo-Tf) with in situ growth of MnO_2_ nanocrystals fabricated by a modified mild biomineralization process. Next, ppIX was chelated with holo-Tf to form MnO_2_@Tf-ppIX nanoparticles (TMP). In a simulated TME in vitro, the responsive release of Mn^2+^ was observed. The r_1_ value increased from 0.78 to 4.16 mmol/(L•s) as the pH decreased from 7.4 to 5.0. Moreover, the r_1_ value [8.90 mmol/(L•s)] was elevated more than 10 times under a glutathione (GSH) concentration of 10 × 10^−3^ mol/L compared with that in the control group without GSH. Furthermore, under a mimic TME with the presence of both H_2_O_2_ and GSH, the r_1_ value increased more than 13 times [r_1_ = 11.07 mmol/(L•s)] that under normal physiological conditions. These results demonstrated the immense potential of TMP as an ultrasensitive contrast agent for T_1_-weighted MRI. In vivo experiments demonstrated a remarkable increase in the MR signal in the TMP group compared with that in the BMP group [referred to as MnO_2_@BSA (bovine serum albumin), with similar appearance, morphology, size, and zeta potential as those of TMP] and the TfR-blocked group. One hour after intravenous injection of TMP, the glioma area showed obviously enhanced imaging performance and reached the highest brightness at 6 h. Therefore, SDT (1.0 MHz, 1.5 W/cm^2^, 50% duty cycle, 3 min) was conducted 6 h after TMP injection. SDT was performed three times at intervals of 3 d, and clear ablation of tumor growth in C6 tumor xenograft mice was achieved. In addition, this type of nanosensitizer also improves BBB permeability with the use of holo-Tf. Holo-Tf showed specific affinity to GBM cells, in which TfR was highly expressed because of their high multiplication rate and iron requirement.

By incorporating MRI guidance and targeted tumor abilities, Zhu et al. [75] constructed multifunctional nanosonosensitizers by incorporating an MnO_x_ component with biocompatible hollow mesoporous organosilica nanoparticles (NPs), followed by chelation with ppIX and cyclic arginine-glycine-aspartic pentapeptide (iRGD, as the targeting peptide). This nanosonosensitizer also improved MRI imaging ability in the presence of H^+^ and GSH in both in vitro and in vivo experiments. Furthermore, the MnO_x_ component could decompose the overexpressed H_2_O_2_ molecules in the TME into O_2_, improving the tumor oxygen level, thereby showing the function of inorganic nanozymes. The RGD could specifically accumulate in the tumor. The comprehensive functions based on the above-mentioned abilities were demonstrated to enhance SDT-induced ROS generation and SDT efficacy.

Additionally, iron oxide (Fe_3_O_4_) NPs have been used as excellent MRI agents for the diagnosis of GBM. The modification of Fe_3_O_4_ NPs with different agents can improve the specificity and sensitivity for the precise location of the GBM. For example, Hu et al. [94] successfully synthesized Fe_3_O_4_@PEI·NHAc-FI-PEG-RGD NPs (PEI, polyethyleneimine; FI, fluorescein isothiocyanate; PEG, polyethylene glycol), which showed r_2_ ultrahigh relaxivity [550 mmol/(L•s)]. The ultrahigh relaxivity may be due to the high magnetic moments of Fe_3_O_4_ NPs fabricated through the mild reaction method and the modification of PEI conferring the appropriate size and accumulation state. This kind of NP can be applied as an efficient nanoprobe for specific MRI of GBM both in vitro and in vivo [95].

More interestingly, nucleic acid-based aptamer—modified Fe_3_O_4_ NPs were constructed by Kim et al. [96] First, carboxylated Fe_3_O_4_ NPs were fabricated using tri-armed carboxyl polysorbate 80 through the nanoemulsion method. Then, the aptamers were conjugated with carboxylated Fe_3_O_4_ NPs through the site provided by carboxyl polysorbate 80. Since functionalized aptamers can bind to vascular growth factor receptor 2 (VEGFR2) overexpressed in GBM angiogenic vessels, aptamer—conjugated Fe_3_O_4_ NPs can effectively assemble in the tumor site and achieve precise imaging of angiogenic vessels. This type of DNA aptamer is a ligand-directed “active targeting” nanomedicine harboring the advantages of high specificity and affinity and avoidance of immunogenicity, which has the potential to be used in nanosonosensitizers in the future [97].

Utilizing the comprehensive ability of multifunctional nanosonosensitizers and potential tumor targeted agents, the optimal time can be determined accurately and the exact tumor margin can be located precisely, which can lead to efficient tumor eradication with minimal morbidities.

## SDT-based combination treatment of GBM

Although SDT has achieved good therapeutic effects, the complex TME (such as hypoxia, high GSH expression, and immune response inhibition) hinders further application of SDT. In addition to the development of multifunctional nanosonosensitizers, combination treatments have emerged as another important strategy for improving the efficacy of SDT. Many combination therapies based on SDT have been extensively explored, such as SDT-PDT, SDT-chemotherapy, SDT-autophagy inhibition, and SDT-thermal treatment.

### SDT combined with PDT

PDT is a type of phototherapy involving light-activated photosensitizers in which ROS are generated to induce tumor cell death. A clinical study that explored photonics-based PDT intraoperatively reported improved survival of patients with GBM [98]. The use of 5-ALA for fluorescence-guided PDT in grades III and IV gliomas has been approved by the FDA [99]. However, due to the limited penetration depth of light (0.5–2.0 mm), deep-seated tumors will not respond well to PDT alone. Fortunately, many photosensitizers, such as porphyrin derivatives, are also sonosensitizers, making it possible to combine both methods to achieve synergistic eradication effects on tumor cells. Furthermore, photosensitizers can be loaded onto sonosensitizers to enhance the enhanced tumor ablation effects. Li et al. [60] reported that the effects of killing C6 glioma cells induced by HMME-mediated SDT (0.5 W/cm^2^, 1 MHz) were enhanced in combination with PDT. The combination group tended to generate more ROS and had a higher apoptotic rate. Using the nanosonosensitizers construction technique, Borah et al. [76] successfully synthesized 3-(1′-hexyloxy) ethyl-3-devinylpyropheophorbide-a (HPPH) and cationic polyacrylamide nanoparticles (PAA‑NMe_3_^+^). Then, HPPH was loaded onto PAA‑NMe_3_^+^ to form a functional nanosonosensitizer. The US can trigger the release of HPPH in a time-dependent manner. These may be related to the inertial cavitation effect. The combination of PDT (fluence: 135 J/cm^2^; fluence rate: 75 mW/cm^2^; 30 min; 665 nm) and SDT (0.5 W/cm^2^, 3.3 MHz, 30 min) can significantly increase the tumor ablation rate of U87-bearing tumor mice from 36% (PDT alone) to 60% after 60 d of therapy. In summary, the application of SDT-PDT, especially the development of novel nanosonosensitizers, can effectively eradicate GBM cells compared with SDT alone. US and lasers can be employed for GBM treatment through the introduction of dual-functional sensitizers (sonic and photo activated). However, most sensitizers are small organic sensitizers that are usually hydrophilic, with low ROS generation efficacy and a lack of tumor-targeting capability. Therefore, future studies should focus on inorganic nanomaterials with good physiological stability, precise tumor-targeting ability, and excellent imaging capability. These nanomaterials can be utilized as nano carriers loaded with organic or inorganic sonosensitizers that can generate ROS under exposure to US.

### SDT combined with chemotherapy

Chemotherapy is a common type of cancer treatment in which chemotherapeutic agents, which are cytotoxic and interfere with cell mitosis, are administered. Many chemotherapeutic drugs are administered intravenously or orally and tend to disperse throughout the body through blood circulation. Normal cells of the bone marrow, digestive tract, and hair follicles that divide rapidly are the most affected, which can cause common side effects, such as immunosuppression, mucositis, and alopecia. Furthermore, due to the heterogeneous TME, chemo-resistance has occurred widely in the treatment process. For GBM, as a result of the presence of the BBB, the number of suitable chemotherapy agents are limited, with only TMZ and bevacizumab recommended by the National Comprehensive Cancer Network (NCCN) guidelines [13]. Integrated sonosensitizers and chemotherapeutic agents can specifically accumulate in tumor sites and the precise release of chemotherapeutic drugs can be achieved by SDT thus reducing the occurrence of conventional side effects. Moreover, SDT can activate the mitochondrial caspase apoptosis pathway, which improves the sensitivity of tumor cells to chemotherapeutic agents. For example, Chen et al. [57] investigated the effects of a combination of TMZ and SDT on C6 glioma cells and the underlying mechanisms. They found that the expression levels of sodium-hydrogen exchanger isoform 1 and matrix metalloproteinase-2 proteins were considerably downregulated in the TMZ + SDT group. Moreover, the expression levels of mitochondrial pathway apoptosis proteins, including Bax, cleaved caspase-3, and Cyt-c, were elevated significantly. These comprehensive effects demonstrated that SDT could improve TMZ resistance and enhance the eradication of GBM cells. Furthermore, inorganic nanosonosensitizers have been introduced. Lee et al. [71] used TiO_2_ NPs conjugated to anti-epithelial growth factor receptor (EGFR) antibody to form antibody–nanoparticle conjugates (ANCs). U87MG (EGFRvIII—negative) and U87MGde 2–7 cells (expressing EGFRvIII) were treated with ANCs. SDT with US (1.0 MHz, duty cycle of 80%, 1.8 W/cm^2^, 1 min) exerted the greatest inhibitory effects on the viability of U87MGde 2–7 cells, with the highest generation of ROS. To compare the efficacy of SDT and PDT in combination with chemotherapy, Madsen et al. [68] investigated ultrasonic and photic activation of AlPcS_2a_ together with the anticancer agent bleomycin (BLM) to treat F98 cells. The author aimed to compare the effects of photochemical internalization (PCI) and sonochemical internalization (SCI) on tumor cells. PCI is a technique that utilizes the photochemical properties of PDT for the enhanced delivery of endolysosomal—trapped macromolecules into the cytoplasm; SCI functions via a similar mechanism. In a previous study, US (0–15 J/cm^2^) was delivered at an interval of 3 min (0–83 mW/cm^2^) to cell cultures, and in the control group, light (670 nm) from a fiber-coupled diode laser (5 mW/cm^2^) was delivered to cell cultures. The researchers found that US using AlPcS_2a_ as the sonosensitizer could improve the BLM colony inhibition rate more efficiently than PCI. Moreover, the improved effects also manifested similar results in three-dimensional tumor spheroids in vitro [69].

With advances in nanomedicine, many nanoplatforms have been designed to encapsulate chemotherapeutic agents, which could help specifically targeted release to the tumors to alleviate the side effects. Wu et al. [77] prepared ROS-responsive IR780/PTX NPs containing ROS-cleavable thioketal linkers (TL) to boost paclitaxel (PTX) release using US. Upon US activation (1 MHz, 0.2–0.4 W/cm^2^, 3 min), IR780/PTX NPs generated large numbers of ROS, inducing apoptosis of U87 cells and promoting PTX release via ROS-sensitive TL decomposition. Furthermore, at the tumor sites, controlled PTX release was achieved by US irradiation (1 MHz, 0.4 W/cm^2^, 3 min). Consequently, the tumor growth was significantly inhibited with no obvious toxicity.

Wang et al. [78] used the encapsulation ability of liposomes and successfully designed a US-activatable porphyrin-phospholipid-liposome (pp-lipo) incorporating doxorubicin (DOX). Approximately 38% and 76% DOX were effectively released after US irradiation of 0.2 W/cm^2^ and 0.3 W/cm^2^ for 60 s, respectively. This process was mediated by ROS generated by SDT, which could induce liposome disruption, resulting in DOX release. The application of DOX-pp-lipo (5 mg/kg) in combination with SDT (1 MHz, 0.3 W/cm^2^, 3 min) could efficiently kill tumor cells in U87 tumor-bearing nude mice through ROS generation, DOX release, and improved vascular permeability.

In summary, SDT-chemotherapy can play a pivotal role in GBM ablation through the effects of combination treatment, including improved chemosensitivity and SDT-induced apoptosis. Recently developed novel nanosonosensitizer platforms can realize the precise release of chemotherapy agents in tumor regions, which can alleviate side effects and achieve efficient tumor ablation. The construction of nanoplatforms incorporating chemotherapeutic drugs and novel sonosensitizers will be a promising SDT method for GBM treatment.

### SDT combined with autophagy inhibition

As an important mechanism of the natural and conserved degradation of cells, autophagy can remove unnecessary or dysfunctional components via a lysosome-dependent regulatory mechanism. Autophagy mainly functions as a tumor suppressor, preventing carcinogenesis in early-stage cancer. It also functions as a tumor promoter, providing nutrients in late-stage cancer [100]. The inhibition of autophagy can effectively enhance the function of anticancer therapies [101]. Previous studies have reported the existence of cross-talk between autophagy and apoptosis [102]. SDT can induce autophagy through a lysosome-dependent process for eradicating impaired organelles (e.g., mitochondria) and proteins [103]. Therefore, it is necessary to introduce autophagic inhibitors to enhance the efficiency of SDT. Qu et al. [79] constructed an intelligent “all-in-one” nanosensitizer containing the autophagy inhibitor-hydroxychloroquine (HCQ), sonoactive chlorin e6 (Ce6), and angiopep-2 peptide-modified liposomes (ACHL) for enhanced SDT. Using rapid BBB opening mediated by UTMD, ACHL could specifically assemble in gliomas. The nanosensitizer could release HCQ and generate ROS simultaneously in the tumor region via secondary US activation. Thus, the SDT induced apoptosis and MAPK/p38-PINK1-PRKN-dependent mitophagy, which could effectively improve the ablation ability of SDT. Furthermore, ACHL-SDT therapy using this nanoplatform significantly inhibited tumor growth and prolonged the OS of orthotopic tumor-bearing mice.

Using inorganic nanosensitizers, Feng et al. [104] successfully fabricated a biomimetic nanoplatform [cancer cell membrane (CCM)-hollow mesoporous TiO_2_ nanoparticles (HMTNPs)/HCQ] incorporating HMTNPs, HCQ, and CCM coating. Possessing homologous targeting ability and biomimetic surface functionalization, CCM-HMTNPs/HCQ could avoid phagocytosis by macrophages, identify the tumor, and accumulate in the tumor efficiently. Then, the released HCQ induced by US stimulation could block the autophagic flux and disconnect the nutrient supply generated from the impaired organelles. Simultaneously, HCQ could improve tumor hypoxia through the vessel normalization effect, which was capable of enhancing the effects of HMTNPs-SDT therapy in an oxygen-dependent manner. CCM-HMTNPs/HCQ could sensitize breast cancer cells to SDT by inhibiting autophagy, which shows promise in tumor treatment. Future studies should refer to this strategy of utilizing inorganic nanoplatforms having the ability to effectively generate ROS. In summary, autophagy exhibited a protective role under SDT-induced oxidative stress, which can compromise the efficiency of tumor eradication. To overcome the difficulty, autophagy inhibitors combined with SDT can serve as a promising strategy for GBM treatment.

### SDT combined with thermal therapy

A previous study reported that moderate thermal effects (42 °C) could significantly enhance the efficacy of PDT by increasing the photosensitization reaction rate and improving tumor hypoxia [105–107]. In light of these findings, Ju et al. [46] investigated whether the combination of SDT and thermal therapy could synergistically contribute to tumor ablation. They found that the group that received SDT plus thermal therapy had significantly high Bax and cleaved caspase-3, 8, and 9 expression levels, which indicated the apoptosis of tumor cells and excellent tumor ablation ability. Subsequently, MRI was introduced to monitor the thermal therapy of the tumor region during SDT and thermal treatment to achieve more accurate control of GBM treatment. Yoshida et al. [49] explored the combination of 5-ALA and transcranial MRI-guided FUS for SDT-thermal therapy via in vitro and in vivo experiments. The combination strategy could induce apoptosis and inhibit tumor growth and progression with minimal injury to healthy brain tissue. To improve the MRI imaging ability, Mn^2+^ ions were introduced by Wan et al. [80], who developed Mn^2+^-conjugated human serum albumin—Ce6 nanoassemblies (HCM NAs). HCM NAs with an average diameter of (75 ± 2) nm were obtained. The HCM NAs exhibited similar ultraviolet—visible absorption spectra as those of free Ce6. Furthermore, the r_1_ values were nearly three times higher than those of Magnevist [4.3 mmol/(L•s)]. U87 glioma cells incubated with NAs for 3 h and irradiated with SDT (1 W/cm^2^, 2 min) at 42 °C tended to induce greater ROS generation, resulting in a higher percentage of apoptosis to achieve more efficient tumor cell eradication. Finally, the combination of SDT (1 W/cm^2^) and moderate thermal therapy (42 °C, 20 min, 1st and 4th day) completely inhibited tumor proliferation in a subcutaneous glioma mouse model, which significantly delayed tumor growth in an orthotopic U87 glioma mouse model after 5 weeks. To further investigate the tumor eradication function of MRgFUS, Wu et al. [81] explored the efficiency of different parameters of transcranial MRgFUS and real-time MRI thermometry monitoring using 5-ALA in combination in a rat brain tumor model. They found that the maximum temperature increase was (2.5 ± 1.0) °C and (3.3 ± 1.2) °C for 32 °C and 37 °C, respectively, with core body temperatures corresponding to 20 min of MRgFUS at an I_SPTA_ of 5.5 W/cm^2^. Both regimens achieved significant inhibition of tumor growth and an increase in OS in an intracranial rat glioma tumor model, and there was no significant difference between the two groups. The author explained that the possible mechanism of SDT at these low intensities is not thermal but mechanical interaction of US and 5-ALA within the tumor, possibly via the bursting of gas bubbles. We believe that the differences in the obtained results may be due to the efficiency of thermal generation of the sonosensitizers. 5-ALA cannot effectively generate sufficient thermal energy compared with HCM NAs. Therefore, by integrating MRI temperature monitoring and SDT-based thermal therapy, this comprehensive platform demonstrated certain advantages. First, with the application of an MRI temperature monitoring system, safety was well guaranteed. Second, the nanosonosensitizers not only achieved SDT effects but also contributed to accurate tumor imaging (e.g., HCM NAs). Finally, the synergistic tumor elimination effect was fulfilled with good biosafety. This platform might be a promising and effective therapeutic strategy for GBM treatment.

## Potential treatment strategies in GBM

With rapid advances in therapeutic methods for tumors, many SDT-based strategies for other types of tumors can hold promise for GBM treatment. Considering the characteristics of GBM, gas therapy, chemodynamic therapy (CDT), and immunotherapy are the potential promising methods that can be used in combination with SDT.

### SDT combined with gas therapy

Gas therapy is a type of therapy in which various gases, including nitric oxide (NO), nitrogen (N_2_), and carbon dioxide (CO_2_), are used. These gases can achieve tumor eradication through a change in the TME. Furthermore, they can enhance the cavitation effects following US irradiation and act as US imaging agents. NO plays a pivotal role in tumor biology. The delivery of high concentrations of NO has been demonstrated to result in nitrosative stress and cause cancer cell apoptosis [108]. Although this type of therapy shows a strong latent capacity for tumor treatment, most NO delivery drugs have a short half-life, low bioavailability, and poor tumor-targeting characteristics, which limit their in vivo efficacy [109]. As the study reported, sphingosine-1-phosphate (S1P) levels increased significantly in GBM, which had specific high affinity with S1P receptors (S1PRs) [110]. Moreover, O2-(2,4-dinitrophenyl) 1-[(4-ethoxycarbonyl) piperazin-1-yl] diazen-1-ium-1,2-diolate (JS-K) is a type of NO prodrug that can generate NO via glutathione S-transferases (GSTs), but its poor water solubility limits its clinical application [111]. Liu et al. [112] successfully designed a liposomal DDS incorporating S1P/JS-K/Lipo, which achieved tumor-targeted delivery and release of JS-K. The authors found that JS-K/S1P/Lipo could pass through the BBB via caveolae-mediated transendothelial transcytosis with inhibition of P-glycoprotein. After overcoming the BBB, JS-K/S1P/Lipo specifically accumulated in glioma cells via the interaction of S1P receptors. Next, NO gas was generated specifically by highly expressed GST in GBM. Nondestructive US imaging was used to observe GST-mediated catalysis of JS-K into cytotoxic micro-sized NO bubbles in the vasculature of GBM tumors. US was used as the only imaging modality in this study, which was sufficient to achieve the ideal tumor eradication function. If SDT is applied as an adjuvant therapy, new multifunctional nanoplatforms incorporating JS-K might be a promising method. Utilizing the advantages of SDT, Feng et al. [113] conjugated tirapazamine (TPZ) into HMTNPs with a reformation of S-nitrosothiol (R-SNO). The HMTNPs acted as sonosensitizers, which can generate ROS following US exposure. Thereafter, the hypoxic environment induced by SDT can activate TPZ to achieve a hypoxia-specific killing function. At the same time, the generated ROS can sensitize R-SNO to release NO. Based on these inspiring developments and further exploration of the mechanism underlying GBM TME, we believe that gas generator agents can show potential in tumor theranostics and therapy in the future.

### SDT combined with chemodynamic therapy

It is generally believed that the TME consists of elevated levels of H_2_O_2_ and is acidic. Employing these characteristics, CDT can be used to induce ROS generation by Fenton or Fenton-like reactions, through which toxic hydroxyl radicals can be produced via the decomposition of H_2_O_2_ in the TME. Circumventing the limited penetration distance of the external stimuli and the subsequent potential injuries to adjacent healthy tissues, CDT shows excellent therapeutic effects and good biosafety. Although SDT can achieve deep tissue penetration, the hypoxic TME limited tumor efficacy by decreasing ROS generation, which depends on the O_2_ level in tumors. Many Fenton and Fenton-like reaction-based nanomaterials are metal-based NPs, e.g., Fe^2+^, Mn^2+^, Cu^2+^, and Ti^3+^ ions [114]. Wang et al. [115] successfully synthesized PEG-TiO_1+x_ NRs possessing the CDT function with highly efficient US-induced ROS generation by using the oxygen-deficient structure of TiO, which could function as charge traps and prevent the recombination of US-induced electron–hole pairs. Meanwhile, because of the presence of Ti^3+^, PEG-TiO_1+x_ NRs could take advantage of H_2_O_2_ to produce highly toxic ^•^OH to achieve efficient tumor ablation. Thus, SDT/CDT with ultrafine PEG-TiO_1+x_ NRs can efficiently kill tumor cells under US irradiation compared with TiO_2_ NPs. To further overcome the protection ability of the TME against ROS damage by abundant GSH, Wang et al. [116] developed vanadium (V)-doped TiO_2_ (V-TiO_2_) nanospindles with the capability of glutathione consumption as a multifunctional inorganic nanosonosensitizer. V-TiO_2_ nanospindles had decreased bandgap compared with TiO_2_ NPs and enhanced the ROS generation rate following US exposure. The doped V also made the V-TiO_2_ nanospindles an efficient Fenton-like agent that improved CDT efficiency. Consequently, V-TiO_2_ nanospindles effectively eliminated the tumors with an improved SDT-CDT combination therapy through the depletion of GSH in the TME, with good biosafety. CDT has the unique advantage of relying on endogenous stimuli in the TME. Because of the heterogeneous characteristics of different tumors, the stimuli are usually limited. Hence, SDT in combination with CDT is a feasible strategy for optimizing the efficiency of tumor eradication. Inorganic sonosensitizers should be given focus owing to their high SDT/CDT efficiency and superior physical and chemical properties. In GBM, the TME is also acidic, which makes it a promising tumor for treatment using the SDT/CDT strategy.

### SDT combined with immunotherapy

Due to various genetic mutations and epigenetic alterations of the TME, immunotherapy for GBM has not achieved an OS benefit thus far. SDT can promote the release of large amounts of tumor-associated antigens from the cell residues of the treated tumor and induce tumor-related immunological responses [117]. Some trials reported that SDT can activate proinflammatory responses, reverse the passive properties of antigen—presenting cells, such as DCs and macrophages, and enhance the tumor infiltration of activated leukocytes [118]. US at an appropriate frequency can further strengthen antitumor immune responses. Therefore, with its diverse biological effects, SDT combined with immunotherapy has the potential to be a powerful clinical approach. Zhu et al. [119] successfully constructed two-dimensional (2D) coordination nanosheets consisting of Zn^2+^ and Tetrakis (4-carboxyphenyl) porphyrin (TCPP) for combined SDT-immunotherapy. The nanosheets showed an obviously higher level of US-induced ROS generation. This type of 2D Zn-TCPP nanosheet had a large surface area; thus, it can be conjugated with cytosine phosphorothioate guanine oligodeoxynucleotide (CpG-ODN) acting as a potent Toll-like receptor 9 agonist. After injection of Zn-TCPP/CpG, SDT can lead to the release of tumor debris, which functions as tumor-associated antigens and induce strong antitumor immune responses. US alone was found to reverse the immunosuppressive status of the TME by activating proinflammatory responses, improving cytotoxic T cell invasion, and inhibiting the activation of regulatory T cells. This type of nanosheet was proven to be extremely effective in activating systemic immune responses to successfully eradicate primary tumors. To mitigate the side effects of a combined checkpoint blockade PD-L1 with traditional clinical therapies, Yue et al. [120] established HMME/imiquimod (R837)@Lip as a nanosonosensitizer incorporating immune-adjuvant and sonosensitizer co-loaded nano-liposomes for combined SDT-immunotherapy. SDT induced the release of tumor-associated antigens harboring vaccine-like functions together with an immune adjuvant, which exhibited an immune response by boosting DC maturation and promoting cytokine secretion. In particular, systemic antitumor immune responses were greatly stimulated with increased tumor-infiltrating CD8^+^ lymphocyte levels after being combined with an anti-PD-L1 checkpoint blockade. This combined therapeutic treatment has been shown not only to suppress primary tumors but also to alleviate tumor metastasis in 4T1 breast cancer and CT26 colorectal cancer murine models. The combined immunotherapy strategy confers long-term immunological memory function to protect against tumor recurrence after the eradication of the primary tumors. This study demonstrated the feasibility of SDT in combination with checkpoint blockade in tumor therapy.

Collectively, the combination of SDT and immunotherapy utilizes nanotechnology to incorporate efficient sonosensitizers with or without an anti-PD-L1 immune checkpoint blockade. On the one hand, SDT alone has the ability to enhance the activation of the immune system and inhibit tumor recurrence and metastasis. On the other hand, STD in combination with an anti-PD-L1 immune checkpoint blockade can realize the precise release of antibodies to improve the immune system and alleviate systemic side effects compared with traditional immunotherapies.

## Conclusions

GBM is a lethal disease with poor prognosis. The past decade has witnessed considerable advancements in GBM treatment, such as multimodal imaging-guided surgery, molecular pathological diagnosis, and machine-learning-based prognostic systems. However, limited improvement in OS has been achieved. Because of the limited permeability of the BBB, most macromolecule-based medicines cannot be delivered efficiently to the CNS, which has made it impossible to cure tumors. Fortunately, TTFields have been developed, which has prolonged the median OS to 22 months. Hence, physical treatment is becoming a promising method for GBM treatment.

SDT has emerged as a new approach to cancer treatment. Utilizing sensitizer agents and low-intensity US, SDT has shown good biosafety and excellent efficiency. Future directions for research should be focused on the development of multifunctional nanosonosensitizers, construction of synergistic nanoplatforms, and development of SDT-based combination methods.

First, conventional sonosensitizers are mostly organic molecules. Although they have good US responses, enduring skin sensitivity, low biostability, and poor tumor specificity limit the efficiency of SDT. Therefore, multifunctional nanosonosensitizers using nanomedicine technology have been developed to overcome the shortcomings. Through modification, the nanosonosensitizers have improved tumor accumulation ability, achieved good biostability, and reduced the cavitation threshold, all of which contribute to more thorough tumor eradication. Moreover, harboring the ability of superior physicochemical properties and good biostability, tremendous development has been made in inorganic sonosensitizers. For example, we investigated Ti-based nanosensitizers in the treatment of tumors and found excellent SDT efficiency with the regulation of TME, simultaneously with CDT ability and GSH depletion. Fortunately, these inorganic sonosensitizers displayed good biosafety and rapid degradation ability in vivo. Therefore, multifunctional nanosensitizers have promising application value in the future.

Second, nanoplatforms including nanosensitizers, tumor-targeted agents, and imaging drugs have been developed to achieve identification of precise tumor location and eradication, which mitigate the side effects caused by conventional drugs that mostly work through blood circulation. Furthermore, combination instruments have been incorporated into the system. For example, UTMD utilizing FUS and MBs to temporarily open the BBB can help more agents enter the CNS to cure the tumor. Moreover, combination MRI devices can assist with tumor imaging and thermal monitoring to improve SDT efficacy.

The use of SDT alone has limitations. Combination treatment strategies should be introduced to overcome these limitations. Combined PDT, chemotherapy, autophagy inhibition, and thermal therapy have been explored for the treatment of GBM, and a synergistic GBM eradication capability was achieved.

These studies were designed based on the advancement of GBM proliferation and invasion mechanisms. Therefore, learning from other SDT-based combination advancements and the characteristics of GBM, we propose possible combination strategies including gas therapy, CDT, and immunotherapy. We believe that SDT should be combined with these new methods to compensate for its disadvantages and maximize tumor eradication effects comprehensively. Moreover, the reviewed studies were mainly conducted in animal models. There is still a long way to go in order to make these possible treatments feasible with acceptable safety in clinical application.

## Data Availability

Not applicable.

## Abbreviations

5-ALA: 5-Aminolevulinic acid
ACHL: Angiopep-2-modified liposomes
AlPcS_2a_: Aluminum phthalocycanine disulfonate
BBB: Blood–brain barrier
BLM: Bleomycin
BMP: MnO_2_@BSA (bovine serum albumin)
CCM: Cancer cell membrane
CDT: Chemodynamic therapy
CNS: Central nervous system
CO_2_: Carbon dioxide
CpG-ODN: Cytosine phosphorothioate guanine oligodeoxynucleotide
DC: Dendritic cell
DDSs: Drug delivery systems
DOX: Doxorubicin
DVDMS: Sinoporphyrin sodium
EGFR: Epithelial growth factor receptor
FDA: Food and Drug Administration
Fe_3_O_4_: Iron oxide
FI: Fluorescein isothiocyanate
GBM: Glioblastoma multiforme
GSH: Glutathione
GSTs: Glutathione S-transferase
HCQ: Hydroxychloroquine
HCM NAs: Manganese ion (Mn^2+^)-chelated human serum albumin (HSA)-chlorin e6 (Ce6) nanoassemblies
HMEE: Hematoporphyrin mono-methil ether
HMTNPs: Hollow mesoporous TiO_2_ nanoparticles
iRGD: Cyclic arginine-glycine-aspartic pentapeptide
JS-K: O_2_-(2,4-dinitrophenyl) 1-[(4-ethoxycarbonyl) piperazin-1-yl] diazen-1-ium-1,2-diolate
KPS: Karnofsky performance score
Lipo: Liposome
MBs: Microbubbles
LITT: Laser interstitial thermal therapy
MGMT: O^6^-methylguanine-DNA methyltransferase
MMP: Mitochondrial membrane potential
N_2_: Nitrogen
NO: Nitric oxide
OS: Overall survival
PAA-NMe^3+^: Cationic polyacrylamide nanoparticles
PCI: Photochemical internalization
PDT: Photodynamic therapy
PEG: Polyethylene glycol
PEI: Polyethyleneimine
pp-lipo: Porphyrin-phospholipid-liposome
ppIX: Protoporphyrin IX
PTX: Paclitaxel
r_1_: T_1_ longitudinal relaxation rate
ROS: Reactive oxygen specie
R-SNO: Reformation of S-nitrosothiol
RT: Radiotherapy
S1P: Sphingosine-1-phosphate
SCI: Sonochemical internalization
SDT: Sonodynamic therapy
SERCA_2_: Sarcoplasmic/endoplasmic reticulum Ca^2+^ ATPase
TCPP: Tetrakis (4-carboxyphenyl) porphyrin
Tf: Transferrin
HPPH: 3-(1′-Hexyloxy) ethyl-3-devinylpyropheophorbide
TL: Thioketal linkers
TME: Tumor microenvironment
TMP: MnO_2_@Tf-ppIX nanoparticles
TMZ: Temozolomide
TPZ: Tirapazamine
TTFields: Tumor treating fields
US: Ultrasound
UTMD: Ultrasound-targeted microbubble destruction
VEGF-A: Vascular endothelial growth factor-A
VEGFR2: Vascular growth factor receptor 2
